# Two-Dimensional Crystals as a Buffer Layer for High Work Function Applications: The Case of Monolayer MoO_3_

**DOI:** 10.1021/acsami.2c09946

**Published:** 2022-08-17

**Authors:** Dorota A. Kowalczyk, Maciej Rogala, Karol Szałowski, Domagoj Belić, Paweł Dąbrowski, Paweł Krukowski, Iaroslav Lutsyk, Michał Piskorski, Aleksandra Nadolska, Patryk Krempiński, Maxime Le Ster, Paweł J. Kowalczyk

**Affiliations:** † Department of Solid State Physics (Member of National Photovoltaic Laboratory, Poland), Faculty of Physics and Applied Informatics, University of Lodz, Pomorska 149/153, 90-236 Łódź, Poland; ‡ Division of Physical Chemistry, Department of Chemistry, Lund University, P.O. Box 124, 22100 Lund, Sweden; § Department of Physics, Josip Juraj Strossmayer University of Osijek, 31000 Osijek, Croatia

**Keywords:** monolayer, 2D, molybdenum oxide, MoO_3_, work function, KPFM, UPS, electrostatic potential, anode material

## Abstract

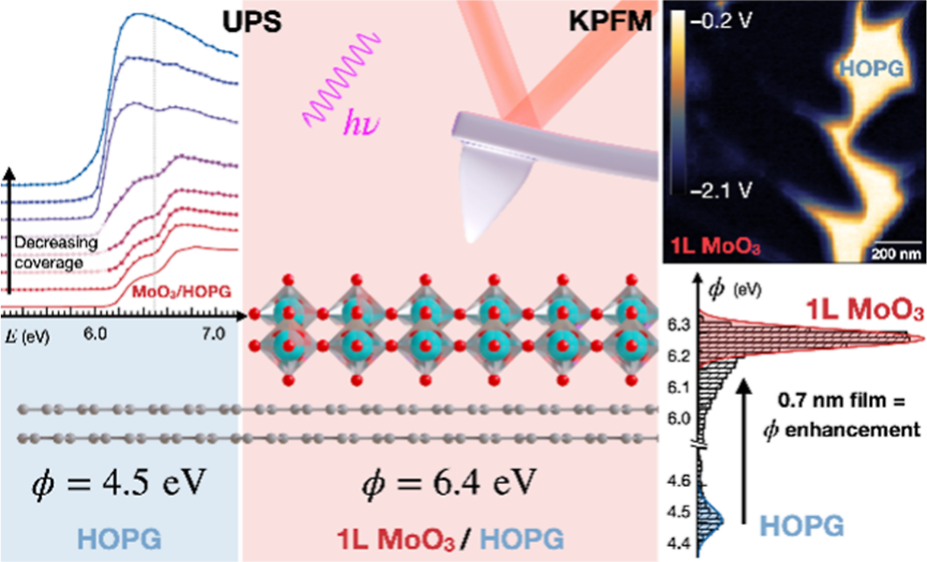

We propose that the crystallinity of two-dimensional (2D) materials is a crucial factor for achieving highly effective work function (WF) modification. A crystalline 2D MoO_3_ monolayer enhances substrate WF up to 6.4 eV for thicknesses as low as 0.7 nm. Such a high WF makes 2D MoO_3_ a great candidate for tuning properties of anode materials and for the future design of organic electronic devices, where accurate evaluation of the WF is crucial. We provide a detailed investigation of WF of 2D α-MoO_3_ directly grown on highly ordered pyrolytic graphite, by means of Kelvin probe force microscopy (KPFM) and ultraviolet photoemission spectroscopy (UPS). This study underlines the importance of a controlled environment and the resulting crystallinity to achieve high WF in MoO_3_. UPS is proved to be suitable for determining higher WF attributed to 2D islands on a substrate with lower WF, yet only in particular cases of sufficient coverage. KPFM remains a method of choice for nanoscale investigations, especially when conducted under ultrahigh vacuum conditions. Our experimental results are supported by density functional theory calculations of electrostatic potential, which indicate that oxygen vacancies result in anisotropy of WF at the sides of the MoO_3_ monolayer. These novel insights into the electronic properties of 2D-MoO_3_ are promising for the design of electronic devices with high WF monolayer films, preserving the transparency and flexibility of the systems.

## Introduction

Work function (WF, Φ), defined as the minimum energy required for an electron to escape from a material surface, is a critical surface characteristic as it determines the interface properties in electronic applications. The introduction of high WF transition metal oxides (TMOs) as buffer layers for anodes in organic electronics has proven to increase efficiency of devices due to securing the energy level alignment.^1,2^ However, further thickness reduction of conventional TMO layers is a difficult task because it has been reported to impact the electronic properties including their WF.^2−6^ Thus, the pursuit of novel designs and alternatives to conventional buffer layer materials is the subject of intense research. In this work, we propose ultrathin (<1 nm) two-dimensional (2D) crystalline films for fine-tuning of the substrate WF, therefore downscaling the thickness of currently used buffer layers. We show that the balance between maximal efficiency and minimal thickness with transparency can be found using an ordered crystallographic structure.

We focus on α-molybdenum(VI) oxide (α-MoO_3_), a van der Waals (vdW), wide-band gap n-type semiconductor with a high WF of 6.9 eV in its bulk phase.^2,6−8^ This value is notably higher than most WFs of typical layered materials such as highly ordered pyrolytic graphite (HOPG) and graphene (4.4–4.5 eV)^2,5^ or MoS_2_ (4.3–5.5 eV).^9,10^ In α-MoO_3_, valence and conduction bands are ∼3 eV apart and the material can be easily doped through oxygen vacancies forming MoO_3–*x*_^7,11−14^ with additional bands below the conduction band minimum and a local decrease in the band gap.^15^ It has been theoretically predicted that α-MoO_3_ retains its bulk properties in the 2D limit, and thus, the electronic structure of 2D α-MoO_3_ is not significantly altered from the bulk,^12,16−19^ including its high WF. This is different to, for example, graphene and monolayer MoS_2_, which may present different electronic properties depending on a number of layers.^10,20,21^

Concerning MoO_3_ and investigations performed by ultraviolet photoemission spectroscopy (UPS), there are many relevant reports on both the thickness dependence and the influence of the substrate on its WF.^2−4,6,22−24^ Much effort has been put into enhancing MoO_3_ WF by increasing film thickness or restoring its high value after air exposure.^2−4,7,8^ The WF of MoO_3–*x*_ films was reported to gradually increase with thickness, for example, at 1.4 nm, it reaches 5.90^3^–6.40 eV,^24^ while final saturation (≤6.80 eV), that is, the highest observed value, on graphene occurs for thicker films of around 4.0–5.0 nm.^2−4^ Although successful, these studies did not investigate the electronic structure dependence on the number of layers of α-MoO_3_ and did not focus on potential improvement by full crystalline coverage essential to accurately describe MoO_3_ monolayer properties. The high WF of 2D-MoO_3_ has not been fully experimentally revealed in part due to the uncontrolled environment and underestimation of results from conventional analytical tools.

The determination of not only the WF value but also its homogeneity across the wider surface is crucial. Despite the importance of UPS, there has been relatively little discussion on how to conduct measurements on patchy samples with laterally heterogeneous WF.^24−28^ At the same time, such heterogeneity is expected in uncoalesced films resulting from island growth, typical of 2D materials. Local WF measurements that can be obtained by Kelvin probe force microscopy (KPFM) are missing for 2D-MoO_3_ in ultrahigh vacuum (UHV). In contrast to previous reports, we combine global WF measurements with a nanoscale KPFM study to link the actual functional properties of MoO_3_ layers with their atomic structure. Such an approach allows us to point the crystallinity and coalescence of MoO_3_ monolayers as important factors for optimization of buffer layers in effective tuning of WF for organic electronic applications.

Our previous work described the possibility of synthesizing 2D nonstoichiometric α-MoO_3_ layers directly on HOPG.^15^ While studying the morphology, chemical, and electronic structure of α-MoO_3_, we also observed a substantial WF decrease in air-exposed samples by UPS, consistent with the literature reports.^7,8^ This work precisely defined the material. However, crucial questions remain open: how the crystallinity of the interface influences the tuning of WF and whether it is an attractive solution for potential applications. This work defines the important and possible general relation between crystallinity of functional materials and optimization of its properties as a buffer layer for energy-level alignment in organic electronics. This study explains what WF the 2D MoO_3_ nanosheets have on a local scale when the UHV environment remains unbroken and how to evaluate WF of uncoalesced monolayers with a global technique accurately.

Herein, we report an investigation of the WF of monolayer (1L) MoO_3_ on HOPG by a combination of two techniques: KPFM and UPS. We start with presenting high crystalline quality of the grown monolayers and continue with local WF measurements over micrometer-size areas, that is, mapping contact potential difference (CPD) by KPFM in UHV. Next to nanoscale imaging, we provide a commentary on UPS measurement of the WF of MoO_3_ monolayers to verify whether this technique is suitable for the determination of WF attributed to 2D islands on a substrate with relatively lower WF. Particular effort is made toward addressing complications associated with double secondary electron cutoff due to insufficient surface coverage. UPS analysis of various 2D-MoO_3_/HOPG lateral area ratios provides information helpful for determining the high WF of one of the materials. Furthermore, density functional theory (DFT) calculations are performed to elucidate the possible underlying mechanism of the influence of oxygen vacancies in MoO_3_ monolayers from two directions: top and bottom face.

Driven by an interest in graphene-based electrodes, the presented 2D-MoO_3_/HOPG system is attractive for fundamental study as a model platform to describe the electronic properties of 2D-MoO_3_/graphene heterostructures. Based on the presented complementary results, we prove that 2D-α-MoO_3_ is an excellent candidate for replacing the commonly used amorphous functional oxide layers with crystalline ones. We indicate the crystallinity as a highly efficient way of tuning the WF of electrode materials simultaneously with keeping the sub-nanometer thickness limit. The proposed approach allows for uniform modification of electronic properties of devices by a single monolayer of high continuity. Because 2D-MoO_3_ can be produced on a centimeter scale (α-MoO_3_),^16,29−31^ our results are potentially easy to scale as needed for industrial applications. In general, our approach carries important implications for characterizing functional interfaces, especially for the purpose of organic electronics. We emphasize that, among global properties and efficiency of the applied materials, the functional layers should be deeply studied on the nanoscale by electronic structure-sensitive techniques, as this is where potential optimization should be looked for. This approach especially paves the way for designing more complex vdW heterostructures.^32,33^

## Results and Discussion

### Monolayer MoO_3_—Morphological Characterization

We synthesized monolayer MoO_3_ directly on HOPG in UHV through thermal evaporation of MoO_3_ powder at elevated substrate temperatures, as described in our previous work.^15^ First, we confirm the growth of a monolayer-thin film. Second, their high crystalline quality is verified. These two features play a crucial role in the electronic properties of MoO_3_/HOPG heterostructures, as described in the following sections.

**Figure 1:**
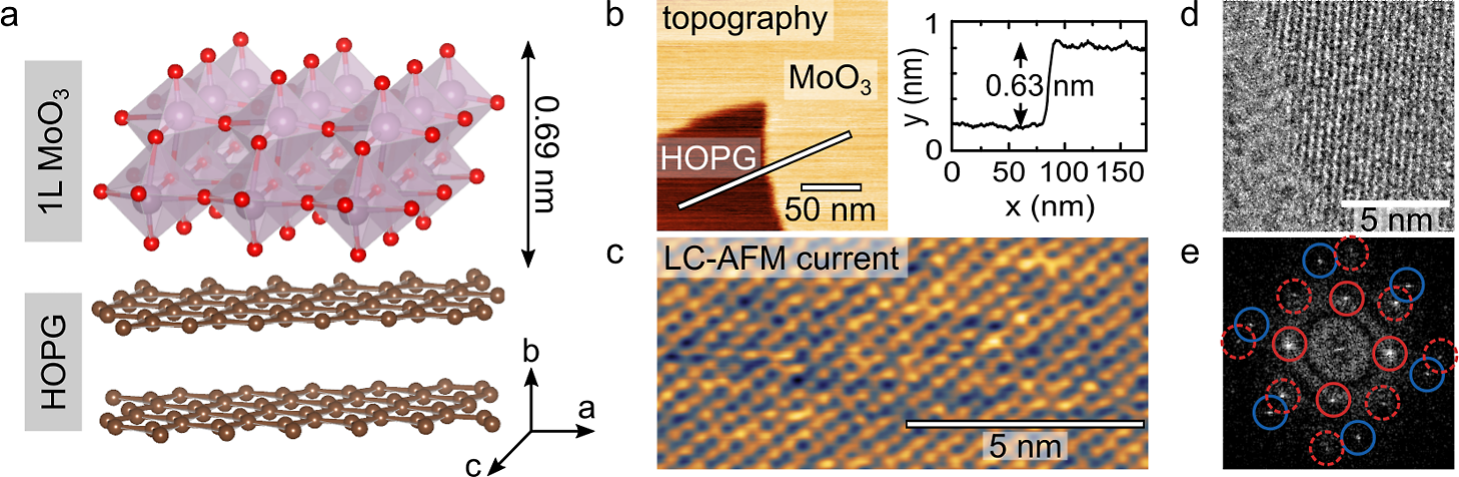
Atomic structure and topographical imaging of the MoO_3_ monolayer (1L) on HOPG. (a) Schematic representation of the MoO_3_ monolayer on HOPG. (b) LC-AFM topography with a cross-section profile taken across the MoO_3_ nanosheet, revealing a monolayer thickness of around 0.63 nm. (c) LC-AFM current: atomic periodicity resolved on the MoO_3_ surface (due to room temperature measurement, the image has been drift corrected; *V* = 5 mV). (d,e) Plan-view HR-TEM image of MoO_3_ flakes on graphene together with calculated FFT; the patterns of MoO_3_ and graphene are circled in red and blue, respectively, while solid and dashed circles indicate first and higher order diffraction spots, respectively.

The α-phase of MoO_3_ is a thermodynamically stable polymorph with a layered structure consisting of chemically bonded octahedral nets aligned in (010) planes linked by weak vdW forces.^12,22,34^ The unit cell of bulk α-MoO_3_ contains two layers separated by a vdW gap, with the following lattice parameters: *a* = 3.96 Å, *b* = 13.86 Å, and *c* = 3.70 Å.^17,18,22,34^ A monolayer of α-MoO_3_ corresponds to half of the unit cell in the *b* direction, which corresponds to a thickness of ∼6.93 Å, and the surface unit cell is defined by *c* × *a*—that is, 3.70 × 3.96 Å^2^. Figure 1a illustrates MoO_3_ forming a monolayer (1L) on a graphene-like substrate, that is, HOPG.

We checked the thickness of the grown MoO_3_ islands using local conductivity AFM (LC-AFM) under UHV conditions without any exposure to air (see the topography in Figure 1b). The thickness of a layer is presented at the profile (Figure 1b), and an extracted step height of around 6.3 Å confirms a monolayer (1L) growth within experimental error of LC-AFM.

LC-AFM is known to resolve atomic contrast in the current image despite a relatively dull tip and allows for identification of atomic periodicity.^35^ The atomic periodicity resolved in the current LC-AFM image in Figure 1c indicates the high crystalline quality of the MoO_3_ monolayer. Note that current imaging is possible due to slight nonstoichiometry of the obtained layer, which induces additional states in the band gap.^15^

In conjunction with the LC-AFM evaluation of the crystalline quality of the 2D MoO_3_ film, high-resolution transmission electron microscopy (HR-TEM) analysis was conducted to assess its crystal structure. TEM-suitable specimens were made by peeling off multilayer graphene flakes with MoO_3_, from the top surface of the bulk HOPG (see the Methods section for more information). The flakes are shown in Figure S1a–d. The atomically resolved HR-TEM section of the image in Figure S1b is magnified and presented in Figure 1d. Together with a fast Fourier transform (FFT) pattern (Figure 1e), HRTEM data suggest the orthorhombic unit cell of α-MoO_3_—the inner diffraction spots, marked solid red—(3.7 ± 0.2) × (3.9 ± 0.1) Å^2^. The outer spots in the FFT, marked blue, are identified as the graphene hexagonal reciprocal lattice. The spots marked with red dashed rings come from higher-order diffraction spots of α-MoO_3_.

For the grown α-MoO_3_ monolayers, the routinely acquired atomic-resolution scans by LC-AFM together with HR-TEM analysis confirm the high crystalline quality. Next, we focus on the characterization of electronic properties—that is, the WF of the 2D-MoO_3_/HOPG system.

### Local Surface Potential on Monolayer MoO_3_ and HOPG—KPFM Imaging

We employed KPFM to measure CPD, which is directly related to WF of MoO_3_ monolayer-thick islands on HOPG. For this purpose, the growth process was stopped before obtaining 100% substrate coverage. The as-prepared samples possess HOPG regions uncovered by MoO_3_, which serve as a reference during WF measurements. The KPFM measurements were performed in UHV to minimize environmental influence. The UHV conditions are vital because the WF of 2D-MoO_3_ is critically influenced by air contaminants: in air, the WF is significantly reduced from 6.9 eV down to ∼5.2 eV for bulk MoO_3_^7,8^ and to ∼4.7 eV for 2D MoO_3_.^15^

**Figure 2:**
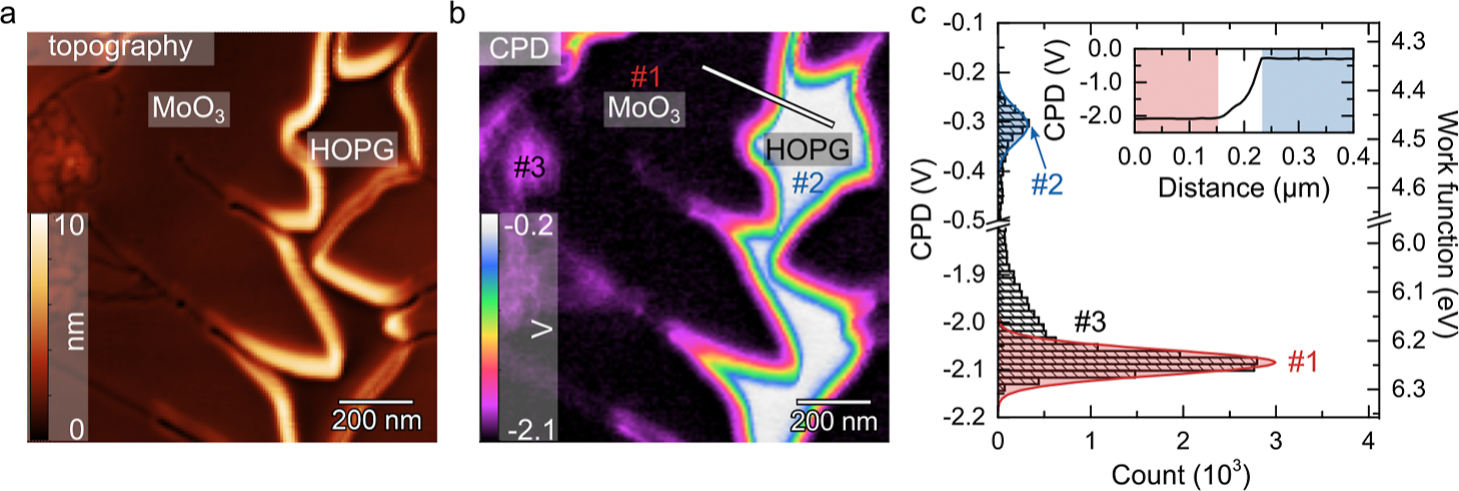
Local WF of the MoO_3_ monolayer (1L) on HOPG: KPFM surface potential measurement in UHV. (a) Topography of 1L MoO_3_ on HOPG, where MoO_3_ edges are artificially lifted. (b) Corresponding CPD map to (a). White/blue color of the CPD signal corresponds to a low WF (HOPG region), whereas pink/black color corresponds to higher WF (MoO_3_ region). (c) Histogram extracted from (b). Lines with shaded areas below represent Gaussian fits for 1L MoO_3_ (red, #1) and HOPG (blue, #2). For details on #3, see the main text. The right axis gives the WF obtained using the calibration on the HOPG reference sample (see Figure 3a). Inset: line scan of the CPD signal corresponding to the line marked in (b) where shaded areas highlight plateaus of CPD for respective materials: MoO_3_ (red) and HOPG (blue).

The topography image (Figure 2a) shows the MoO_3_ monolayer grown on HOPG—a typically shaped island of MoO_3_ consisting of multiple domains.^15^ Examination of the 1L MoO_3_ flake boundaries shows an artificial edge height of around 10 nm; it is an artifact due to uncompensated electrostatic tip–sample interaction and thus appears in regions where the tip apex interacts with both materials having a significant difference in CPD.^36^ In this image, one can also recognize that the MoO_3_ flake is divided by trenches associated with leaf-like structures on its edges. This division indicates the presence of strong perimeter diffusion toward the tip of growing leaves and some crystallographic misalignment preventing the healing of the trenches. Finally, it is worth pointing out that MoO_3_ tends to form a complete monolayer first, and subsequent deposition leads to the formation of islands of multilayers.^15^ However, even in uncoalesced monolayer films, some multilayers can be found (∼100 nm of lateral dimension). This observation indicates lower adsorption energy of incoming clusters on edges than on terraces.^15^

The corresponding CPD map in Figure 2b shows a clear contrast between the HOPG substrate and MoO_3_. The white color on the HOPG surface is attributed to a high CPD signal, which corresponds to low WF values, while the pink/black color at the MoO_3_ monolayer indicates lower CPD and higher WF. The line profile taken over the MoO_3_/HOPG surface in the CPD map (inset in Figure 2c), as indicated in Figure 2b, exhibits two plateaus. The WF difference between the HOPG and 2D-MoO_3_ extracted from the profile is negative (ΔCPD_MoO_3__/HOPG = −1.80 V), indicating that the WF of the MoO_3_ is higher than that of HOPG.

A histogram extracted from the CPD map is given in Figure 2c. For simplicity, the scale on the right represents WF values as calibrated using WF of HOPG. For quantitative evaluation, data were fitted by two Gaussian distributions, from which mean CPD and full width at half-maximum (FWHM) are determined for both MoO_3_ and HOPG: CPD_MoO_3__ = −2.10 V (peak #1) and CPD_HOPG_ = −0.30 V (peak #2), where FWHM for both materials is at the same level of 0.03 V. This leads to the surface potential difference between the 1L-MoO_3_ islands and the HOPG background of ΔCPD_MoO_3_/HOPG_ = −1.80 ± 0.05 V. We also reference CPD measurements to a known WF, which allows calculation of WF of 2D-MoO_3_. Thus, with the calibration to the WF value of HOPG, Φ_HOPG_ = 4.50 ± 0.10 eV (see UPS results in the next section), and the MoO_3_ 2D-islands exhibit WF as high as 6.30 ± 0.12 eV.

In regard to WF homogeneity of the MoO_3_ monolayer, it is also worth noting how growth defects affect the WF. Some topographic protrusions can be seen in Figure 2a on the left-hand side. On the corresponding CPD map in Figure 2b, a faint contrast pointing toward slightly lower WF can also be seen besides grain edges, which coincides with the topographic protrusions in the upper left corner. This variation is quantitatively shown in the histogram (Figure 2c) at count columns marked #3 (at −2.00 V) next to the #2 peak. While we assign peak #2 to the area of the smooth MoO_3_ monolayer, #3 can be assigned to the lower WF at topographic defects. See also Figure S2 with histograms considering selected areas over the CPD map. Note that the surface potential differences at MoO_3_ are within approx. 0.10 V over areas with structural defects.

Furthermore, we observed that the high WF is achieved independent of MoO_3_ grain size. For small grains and big grains, a plateau of CPD is observed at the same level—see Figure S3 with multiple grains of various sizes.

Additionally, we support our CPD measurements independent of topographical height variations. Figure S4 shows HOPG steps of height of about 11 nm; the corresponding CPD map displays homogenous CPD distribution, that is, both HOPG terraces are covered by MoO_3_, and are indistinguishable on the CPD maps.

### WF of Monolayer MoO_3_/HOPG—UPS Characterization

**Figure 3:**
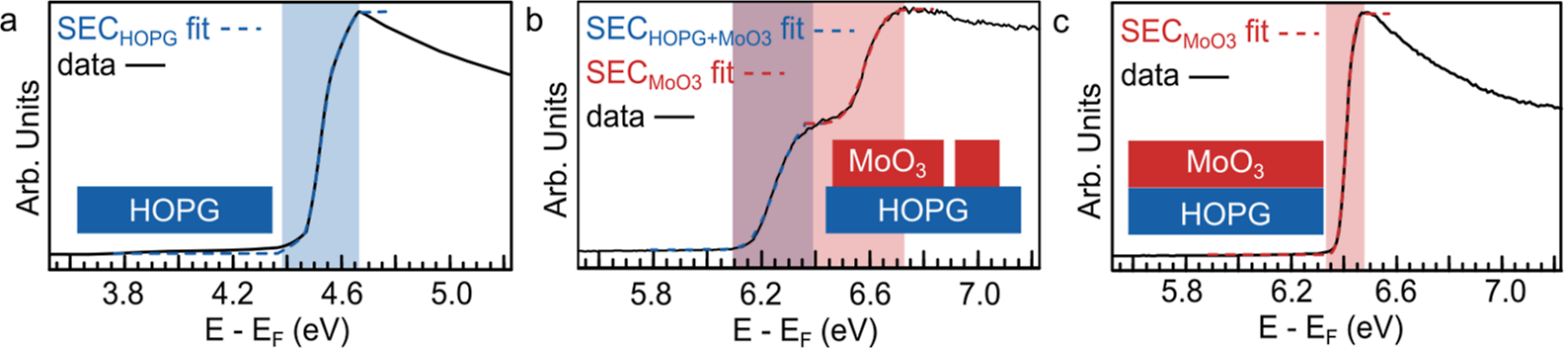
WF of the MoO_3_ monolayer on HOPG. He I UPS spectra of SEC regions: (a) reference spectra of UHV-cleaved pristine HOPG (blue), (b) partially covered HOPG with islands of monolayer MoO_3_, and (c) monolayer film of MoO_3_ (red). The colored dashed curves indicate the fits of SECs, while the shaded areas highlight SEC width. Insets: Schematic illustrations of a MoO_3_/HOPG sample coverage.

Next, we use UPS to confirm the local WF with a global method and show how UPS determines WF of the uncoalesced 2D-MoO_3_ film. Here, we characterize samples of fractional coverage and the coalesced 1L MoO_3_ film. Figure 3 shows three steps in the evolution of the UPS spectrum at the low-kinetic energy region: from pristine HOPG, although partially covered substrates by islands of MoO_3_ monolayers, and complete coverage of MoO_3_. The WF is determined from the intersection of linear extrapolation of an edge of secondary electron cutoff (SEC) with the background;^5,15^ the *x*-axis, that is, photoelectron energy, is calibrated to the Au(111) Fermi level (energy—*E*_F_) such that the energy indicated by SEC corresponds to the WF. For clarity, the insets illustrate the coverage of MoO_3_ on the HOPG substrate.

We begin by measuring the WF of our reference sample—the UHV-cleaved pristine HOPG substrate. The UPS spectrum in Figure 3a presents an SEC, from which one can read a WF of 4.50 ± 0.10 eV. This value agrees with literature values given in the range of 4.40–4.60 eV.^15,37,38^

With fractional coverage by MoO_3_ monolayers on top of HOPG (Figure 3b), the SEC significantly shifts to higher energy, indicating a higher WF. However, the high-intensity peak corresponding to the SEC consists of two components observed at 6.15 ± 0.15 and 6.50 ± 0.15 eV. We assign the first SEC at low energy (highlighted purple) to an apparent WF value of both: uncovered HOPG patches (blue) and top MoO_3_ islands (red), while the second SEC at high energy (highlighted red) corresponds to the MoO_3_ monolayer only. The double SEC can be explained by electrostatic potential distribution above a two-component surface. The electrostatic potential of MoO_3_, that is, the high WF patches, creates an additional energy barrier for photoelectrons emitted from uncovered HOPG, that is, the low WF patches. The explanation of the observed effect is well described by Schultz et al.^25^ More on this topic will be covered at the end of this section. Details on fitting distinct SECs as step edges in CasaXPS are presented in Figure S5.

Finally, after depositing a coalesced MoO_3_ monolayer film (Figure 3c), the SEC interpretation is again straightforward, and the WF of MoO_3_ approaches 6.40 ± 0.10 eV in agreement with our KPFM measurements. This confirms that the high-energy SEC in Figure 3b provides an accurate measure of 2D MoO_3_ WF (see also Figure S6 with overlaid spectra for direct comparison). The WF values we obtained are summarized in Table 1 together with literature values for comparison.

**Table 1:** Comparison between the Obtained Results and the Literature Values of MoO_3_, HOPG, and Graphene WFs

material	sample	WF (eV)	method	references
MoO_3_	1L MoO_3_(010) on HOPG(0001)	6.30 ± 0.12	KPFM in UHV	this work
	1L MoO_3_(010) on HOPG(0001)	(6.40–6.50) ± 0.10	He I UPS	this work
	1L MoO_3_(010)	6.67	DFT	this work
	1L MoO_2.875_ (side with O vacancy)	6.36	DFT	this work
	1L MoO_2.875_ (side without O vacancy)	6.72	DFT	this work
	air-exposed 1L MoO_3_(010) on HOPG(0001)	4.70 ± 0.10	He I UPS	(39)
	amorphous MoO_2.86_ of a nominal thickness of 1.4 nm on 1L graphene on Cu	5.92	He I UPS	(3)
	2 nm coalesced film of reduced MoO_x_ on transferred 1L graphene on Si/SiO_2_	6.42	He I UPS	(2)
	bulk MoO_3_	6.90	He I UPS	(2), (6)–^8^
	air-exposed bulk MoO_3_	5.20	He I UPS	(7), (8)
HOPG	UHV-cleaved HOPG(0001)	4.50 ± 0.10	He I UPS	this work
graphene	2L graphene on SiC(0001)	4.50	He I UPS	(5)
	UHV-annealed (350 °C) 2L graphene on SiC(0001)	4.19	He I UPS	(5)

It should be noted that the shape of the SEC region is a measure of both uniformity of WF and surface cleanliness.^27,40^ Again, the width of SEC increases with inhomogeneity of the WF within the probed area: the width of double SEC of fractional coverage by 2D-MoO_3_ (Figure 3b) is wider than single SEC of the coalesced film (Figure 3c). The monolayer of MoO_3_ features homogenous WF (the SEC width, i.e., FWHM = 0.05 eV), as shown in Figure 3c. Moreover, since air-derived contaminants lower the WF of clean MoO_3_,^15^ the absence of any low kinetic energy tail indicates high surface cleanliness (Figure 3c).

**Figure 4:**
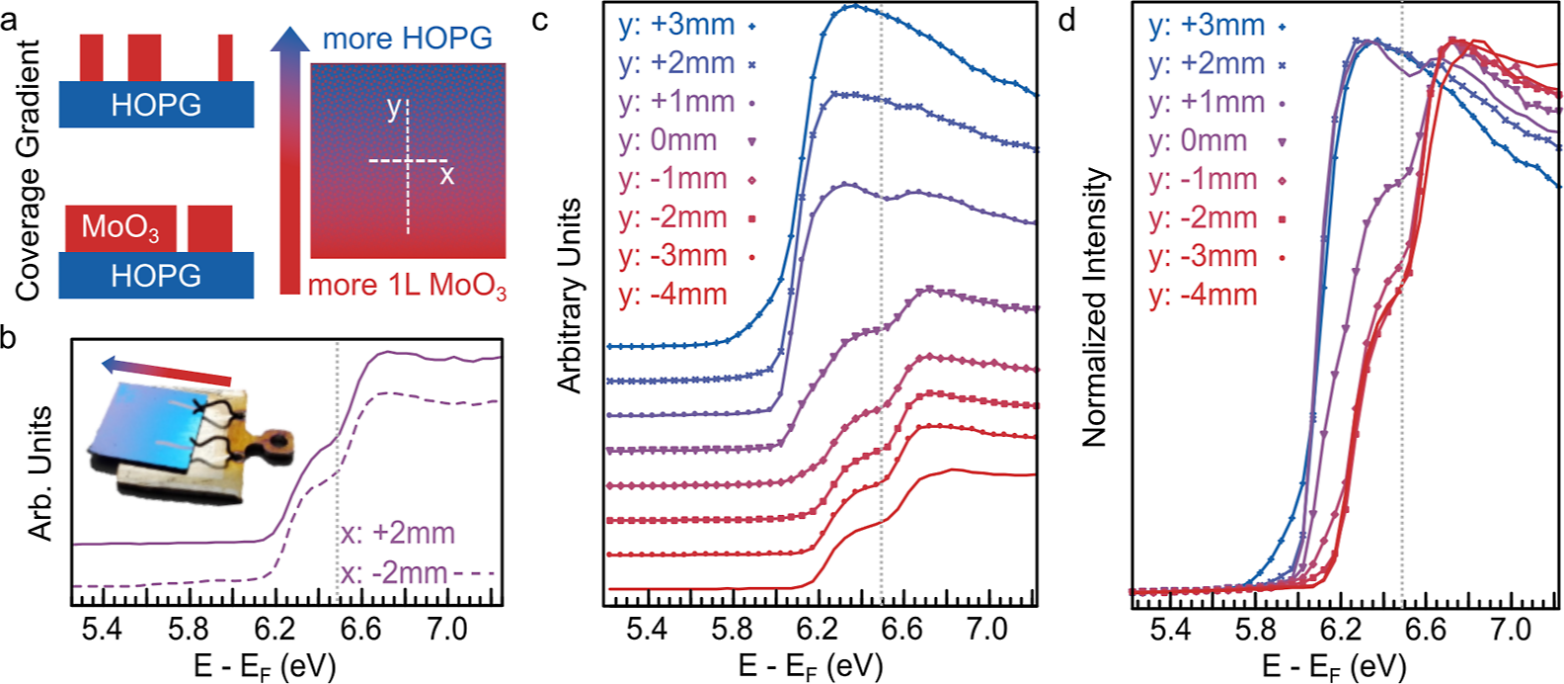
Series of He I UPS spectra of a multicomponent surface—heterogeneous WF—of different area ratios of HOPG and monolayer MoO_3_ patches. (a) Schematic illustration of a patchy MoO_3_/HOPG sample structure; UPS measurements were taken along marked lines: (b–d) SEC region with a range of spectra taken at various points across the sample in intensity arbitrary units (b,c) and normalized intensity (d). The vertical lines indicate the positions of high-energy SECs, corresponding to the WF value of MoO_3_. The inset in (b) represents the gradient of MoO_3_ surface coverage from low to high—additional photography was taken on the SiO_2_ substrate for visualization.

At this point, it is worth discussing the interpretation of UPS for studies of uncoalesced films or 2D flakes with higher WF than their substrate. UPS has become, in recent years, a popular technique for measuring the efficacy of WF tailoring for photovoltaic and light-emitting devices.^2−5,8,24^ Our setup seems to be a perfect system to discuss the limitation of this technique for nonhomogenous systems. As mentioned earlier, in the literature, some efforts focus on patchy samples with laterally nonhomogeneous WF, where patchiness derives from dirt or regular patterning.^24−28^ Here, we present a real-life example where area of <1 nm-thick patches is irregular and depends on the morphology of the substrate because MoO_3_ tends to nucleate at terrace edges of HOPG. The topography of our intentionally prepared test sample can be described as randomly distributed MoO_3_ islands, which take different shapes, but it is worth noting that there is a gradient of surface coverage from dense on one side to scarcer on the other (Figure 4a). For visualization of the coverage gradient, additional photography was taken on an interference pattern of the SiO_2_ substrate with the MoO_3_ thin film—see the inset in Figure 4b. Due to this gradient, we can investigate different coverages of MoO_3_ over the underlying HOPG within the probed area by varying the position for the UPS measurement across a sample with the uncoalesced monolayer film. We took a range of spectra at various points (see lines in Figure 4a) to show how acquired UPS spectra can vary under such conditions. As expected, the UPS spectra are consistent within the horizontal line, where coverage is similar—see Figure 4b. On the other hand, when the MoO_3_/HOPG area ratio varies—vertical line in Figure 4a—the UPS spectra significantly change. Figure 4c presents the SEC region of that series of spectra, while Figure 4d replots them with normalized intensity to highlight the positions of high-energy SECs, corresponding to the WF value of MoO_3_, which are marked by dotted vertical lines. The shift in the low-energy SECs is clear (about up to 200 meV), and the change in SEC shape, width, and relative intensities of distinct SECs is believed to depend on the detail of the MoO_3_/HOPG morphological arrangement within the probed area. Regarding the latter, note that if the area covered by MoO_3_ is insufficient to contribute and build up a distinct high-energy SEC feature, a spectrum reveals only one SEC feature—see the top blue spectrum in Figure 4c. In this case, the pure MoO_3_ contribution is hidden, and the single broad SEC reveals a WF higher than in bare HOPG and lower than that of MoO_3_ due to a higher electrostatic barrier for photoelectrons coming from HOPG as a result of the presence of MoO_3_ islands. To discuss the usability of UPS for WF determination of 2D islands, some useful information can be deduced from these measurements, as listed below:

(I) for different coverages of the sample (in the form of 2D-MoO_3_ islands), the high-energy SEC (if visible) is uniformly^25^ located in our case at ∼6.50 eV; thus, it is determined as the absolute value of WF of a higher WF material, that is, MoO_3_;
(II) the energy of the low-energy SEC shifts; thus, it is dependent on the area ratio^25^ between the substrate and high WF material (here, exposed HOPG and MoO_3_) because the position of low-energy SEC is governed by electrostatic potential distribution above a two-component surface;
(III) with the low-energy SEC shifting toward lower energies, the relative heights of the peaks change, that is, the contribution from HOPG increases, and thus, the intensity of the low-energy SEC increases; likewise, the overall intensity of a spectrum in the SEC region also increases^25^—the same variation in SEC was found for patches composed of ITO and Au by Sharma et al.;^26^ and
(IV) to observe a distinct high-energy SEC, a minimum area of a high WF material is required. Below this coverage threshold, the UPS measurements do not allow for clear conclusions about effective electronic properties of a material.

### DFT Calculations of Electrostatic Potential of Monolayer MoO_3_

The experimental finding of the WF of pristine monolayer α-MoO_3_ grown on HOPG approaches 6.40 eV, which is lower than bulk α-MoO_3_ (6.9 eV^2,6−8^). The theory predicts that the electronic structures of a monolayer and bulk α-MoO_3_ do not alter significantly.^12,16−19^ Nevertheless, the actual WF is sensitive to many factors, including the underlying substrate and stoichiometry.^6,36,41^ The latter is known to influence the WF value of TMOs.^42−44^ Moreover, the possible LC-AFM current image shown in Figure 1b may indicate a slight nonstoichiometry in our films. Even a slightly nonstoichiometric α-MoO_3_ monolayer is conductive; thus, it may not reach the highest possible WF for the bulk material. Here, we present theoretical predictions of WFs of stoichiometric and nonstoichiometric monolayers.

**Figure 5:**
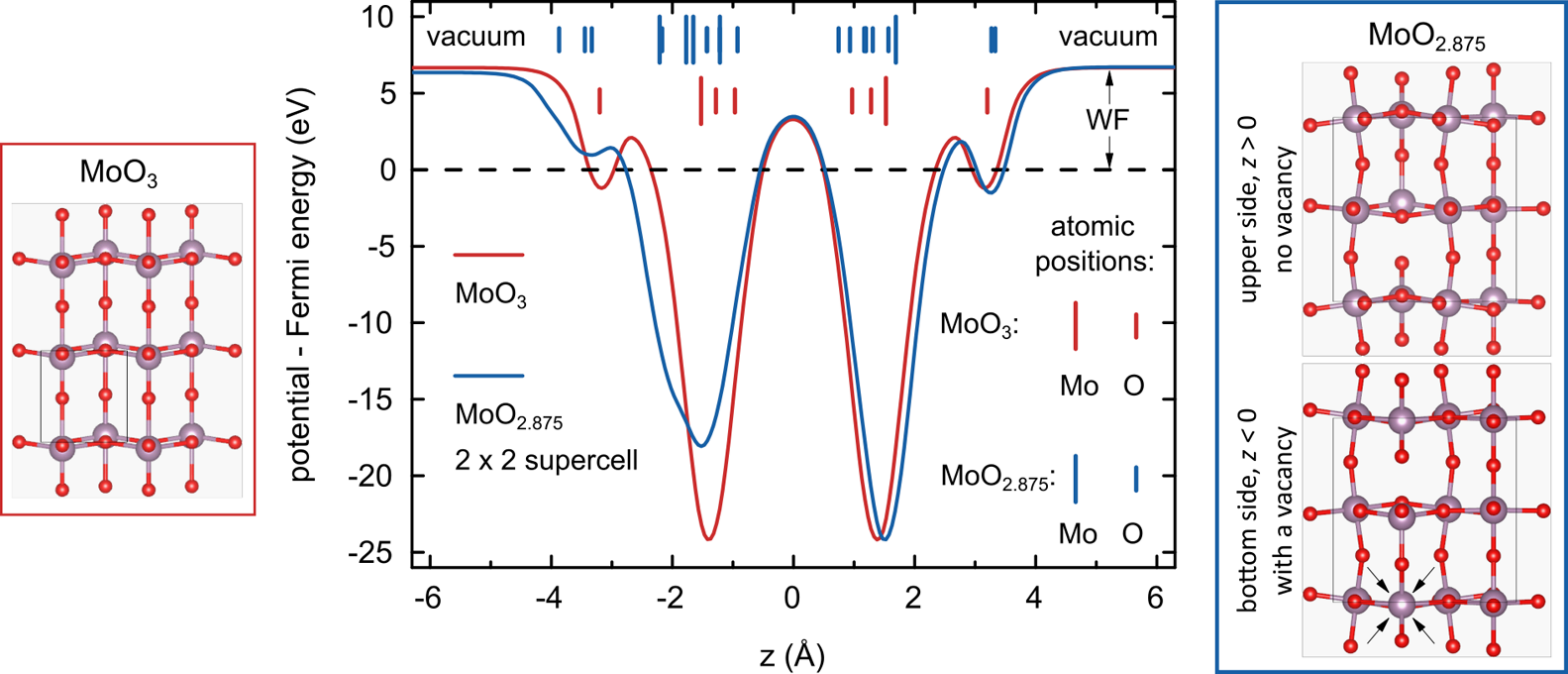
DFT calculations of electrostatic potential of monolayer MoO_3_(010): stoichiometric (red) and defective (blue). Plane-averaged electrostatic potential energy across the pristine MoO_3_- and MoO_2.875_-vacuum interface, that is, as a function of position in the *z*-direction, wherein the energetic difference between the Fermi (set at 0 eV, dashed line) and vacuum levels is the WF. The top view of atomic structures of considered monolayers is given; for MoO_2.875_, two sides are distinguished: upper side of the monolayer with no vacancy and the bottom side with the O vacancy defect, which is pointed out by four arrows on the structure.

The WF can be estimated by the DFT calculations, based on electrostatic potential across the interface of a material and vacuum. The calculations were carried out for two monolayer structures: fully oxidized MoO_3_ and reduced MoO_2.875_ (with a single oxygen vacancy per 2 × 2 supercell, located in the bottom layer; see Figure 5).

The electrostatic potential predicted by our DFT calculations averaged over the monolayer (*xy*) plane is plotted in Figure 5 as a function of the *z* coordinate which is orthogonal to the monolayer plane (along the *b* axis), that is, across the monolayer–vacuum interface. The zero value of the *z* coordinate is selected in the plane in between Mo atoms (precisely at the average of *z* coordinates of all the atoms for the studied structure). The location of the atoms along the *z* axis in the calculated unit cells is marked in the plot with vertical lines (see Figure 5). For the MoO_3_ monolayer without defect (marked red), the distance dependence of the electrostatic potential is fully symmetric with respect to the middle plane. Two deep potential minima correspond to Mo atoms and pairs of nearby O atoms, whereas much shallower minima mark the positions of outer O atoms in the structure. The WF value amounts to 6.67 eV.

The symmetry mentioned above is perturbed for the MoO_2.875_ monolayer (see the blue plot in Figure 5) with a single O vacancy. For the upper side with no vacancy (*z* > 0), the increase in the interatomic distances along the *z* axis is the only noticeable feature (see the shifted minimal positions and atomic coordinates). The predicted WF value on the defect-free side is only slightly increased with respect to the stoichiometric MoO_3_ and amounts to 6.72 eV. At the bottom side with the vacancy defect (*z* < 0), the atomic positions are much more perturbed, as visible in shallow and manifestly asymmetric potential minimum, also shifted due to increasing interatomic distances. The WF predicted at the defect side is reduced to 6.36 eV. Such reduction suggests that our experimental data are affected by imperfections in the stoichiometry of MoO_3_. Our 2D-MoO_3_ was likely slightly oxygen-deficient, in line with the previous reports on thermally evaporated MoO_3–*x*_.^8,15,45^

So far, we have focused on the WF estimated from the constant values of the planar average electrostatic potentials at a considerable distance from the surfaces. We additionally refer the reader to Figure S7, which shows WF maps for both structures in certain *z* distances over the surfaces—it is particularly interesting how WF is distributed over the MoO_2.875_ monolayer from each side. These WF maps theoretically predict the ultimate resolution KPFM measurement, which cannot be achieved in real-life experiments.

## Conclusions

The MoO_3_ monolayer films presented here exhibit electronic properties, that is, high WF, similar to bulk MoO_3_, while being <1 nm thick and quasicontinuous. We achieved a WF of approximately 6.40 eV for the crystalline MoO_3_(010) monolayer on HOPG(0001); this value was reproducible across the samples, provided that UHV conditions were used for both their growth and characterization. The DFT calculations predict the significant influence of nonstoichiometry on the WF value and indicate that the oxygen vacancies on one side of the MoO_3_ monolayer cause the variation in WF of an opposite sign for each face.

In contrast to the existing reports, we emphasize that crystalline 2D-MoO_3_ guarantees high WF, and in this regard, the material does not require thicker layers but coalesced monolayers. With maturing synthesis of continuous monolayer films, which tend to wet graphene-like substrates, 2D MoO_3_ becomes a great candidate for a monolayer buffer layer for WF enhancement.

Our observations not only address the critical issue of the WF for 2D MoO_3_ under UHV conditions but also represent a real-life example of how the commonly accessible UPS apparatus can be used to determine WF attributed to 2D islands or flakes on a substrate with lower WF. Thus, our study showcases a simpler way to obtain a WF of 2D nanomaterials using UPS only, without the need for additional characterization techniques, which may also act as a tool supporting future investigations. These observations carry important implications for the characterization of 2D materials beyond MoO_3_ and the design of vdW heterostructures for energy-level alignment with graphene-like substrates.

## Methods

### Monolayer MoO_3_ Synthesis

The synthesis was the same as that used in ref (15), namely, films of α-MoO_3_ were thermally evaporated on heated to 220 °C HOPG(0001) substrates (ZYA grade) from the powder source of MoO_3_ (Sigma-Aldrich). Freshly deposited films were loaded into a vacuum suitcase and transferred to the analysis chamber of an ultrahigh vacuum (UHV) Multiprobe P (Scienta-Omicron) system with a base pressure of 5 × 10^–10^ mbar, where all UPS and AFM measurements were performed at room temperature.

### UPS Characterization

A hemispherical energy analyzer Phoibos 150 (SPECS), with a 2D-CCD detector, was used for photoelectron spectroscopy measurements. UPS studies were performed with the helium I line (21.22 eV) of VUV Source HIS 13 (Focus GmbH) on samples perpendicular to the analyzer and with an applied bias of −3.1 V.^27^ At a pass energy of 5 eV, the analyzer offers an energy resolution of ∼130 meV as determined from the Fermi edge of Au(111) for the settings used in this study (step size of 0.03 eV). The calibration was conducted at the Fermi level of Au(111).

Analysis of the photoemission spectra was performed using CasaXPS software. The WFs were derived from the intersection of linear extrapolation of a steep edge of SEC with the background, that is, edge-up background type provided in CasaXPS (see Figure S5).

### AFM Characterization

Local-conductivity AFM (LC-AFM) and frequency-modulated KPFM (FM-KPFM) experiments were performed with an Omicron Matrix system and a Nanonis controller (SPECS) with bias applied to the tip during the measurement. CPD maps were obtained simultaneously with topography noncontact-AFM (nc-AFM) images. The probes were highly doped silicon NANOSENSORS PPP-NCH with a nominal resonance frequency and force constant of 330 kHz and 42 N/m, respectively, with an AFM tip radius of curvature of <10 nm when first used. The scanning conditions were chosen to highly reduce the tip–sample distance while maintaining the stability of measurement, and the frequency shift setpoint near −20 Hz was used. This allows us to improve spatial resolution of CPD and minimize topographic artifacts at MoO_3_ edges, which were a significant issue due to the very large WF difference between the materials.

The contrast of a CPD map is directly related to WF differences on the surface. The WF of MoO_3_ was calculated with respect to the WF of HOPG measured using UPS (HOPG was cleaved in UHV before the measurement; see Figure 3a) according to the following equation: Φ_MoO_3__ = Φ_HOPG_ + *e* × (CPD_HOPG_ – CPD_MoO_3__), where *e* is the elementary charge. The KPFM images were processed using Gwyddion software.^46^

### TEM Measurements

TEM imaging was performed at the National Center for High Resolution Electron Microscopy (nCHREM) at Lund University. The MoO_3_/HOPG samples were a few weeks old and subjected to exposure to an inert (Ar) atmosphere for transportation and laboratory air (typically below 5 min) to prepare TEM-suitable specimens. The MoO_3_/HOPG flakes were peeled off and mounted on a TEM grid (lacey formvar-carbon film on 200 copper mesh, Ted Pella, Redding, USA). The imaging was performed in a JEOL JEM-2200FS TEM at 200 keV in a low-dose mode (total dose < 200 e Å^–2^), thus minimizing possible beam damage. The images were recorded on an F416.0 camera (TVIPS), using Serial EM software.^47^

### Computational Methods

The *ab-initio* calculations were performed using the QUANTUM ESPRESSO suite, implementing DFT^48^ using the plane-wave pseudopotential method.^49,50^ The scalar relativistic pseudopotential approach with the projector augmented wave method^51^ was used, with the Perdew–Burke–Ernzerhof exchange correlation functional^52^ accepted. The empirical van der Waals correction^53^ and the dipole correction^54^ were taken into account. The 2D layers were modeled in slab geometry; the calculations involved a monolayer of MoO_3_ and a monolayer of MoO_2.875_ modeled using a 2 × 2 supercell with a single O atom removed from the lower atomic plane. In all the calculations, the total cell size in the direction perpendicular to the layer was set to 50 Å to ensure sufficient vacuum. For structural optimization, the variation of both in-plane lattice constants and all the atomic positions was allowed, ruled by the Broyden–Fletcher–Goldfarb–Shanno (BFGS) quasi-Newton algorithm, based on the trust radius procedure.

All the structural relaxation and self-consistent calculations were based on 24 × 24 mesh of *k*-points. For the monolayer MoO_3_ structure, the convergence criteria were as follows: 10^–11^ Ry for energy and 10^–9^ Ry/Bohr for force. For MoO_2.875_, the convergence criteria were as follows: 10^–6^ Ry for energy and 10^–4^ Ry/Bohr for force.

## Supporting Information Available

Additional HR-TEM images; KPFM images with detailed histogram analysis; UPS spectra of MoO_3_/HOPG including fitting of step edges at SECs and direct comparison of the discussed spectra; and DFT calculations of electrostatic potential maps of MoO_3_ and MoO_2.875_ monolayers (PDF)
