# Supplementary material for: Two-Dimensional Crystals as a Buffer Layer for High Work Function Applications: The Case of Monolayer MoO3
Source: ACS Appl Mater Interfaces. 2022 Aug 17;14(39):44506–15. doi: 10.1021/acsami.2c09946 (PMC9542700; doi:10.1021/acsami.2c09946)
Supplement: Supplementary file 1 — am2c09946_si_001.pdf [file am2c09946_si_001.pdf]

Supporting Information

Two-Dimensional Crystals as a Buffer Layer for High Work Function Applications: the Case of Monolayer MoO_3

Dorota A. Kowalczyk[1]*, Maciej Rogala[1], Karol Szałowski[1], Domagoj Belić[2,3], Paweł Dąbrowski[1], Paweł Krukowski[1], Iaroslav Lutsyk[1], Michał Piskorski[1], Aleksandra Nadolska[1], Patryk Krempiński[1], Maxime Le Ster[1], and Paweł J. Kowalczyk[1]*

[1] Department of Solid State Physics (member of National Photovoltaic Laboratory, Poland), Faculty of Physics and Applied Informatics, University of Lodz, Pomorska 149/153, 90-236 Łódź, Poland

[2] Division of Physical Chemistry, Department of Chemistry, Lund University, P.O. Box 124, 22100 Lund, Sweden

[3] Department of Physics, Josip Juraj Strossmayer University of Osijek, 31000 Osijek, Croatia

E-mail: dorota.kowalczyk@uni.lodz.pl; pawel.kowalczyk@uni.lodz.pl

Transmission electron microscopy (TEM) characterisation

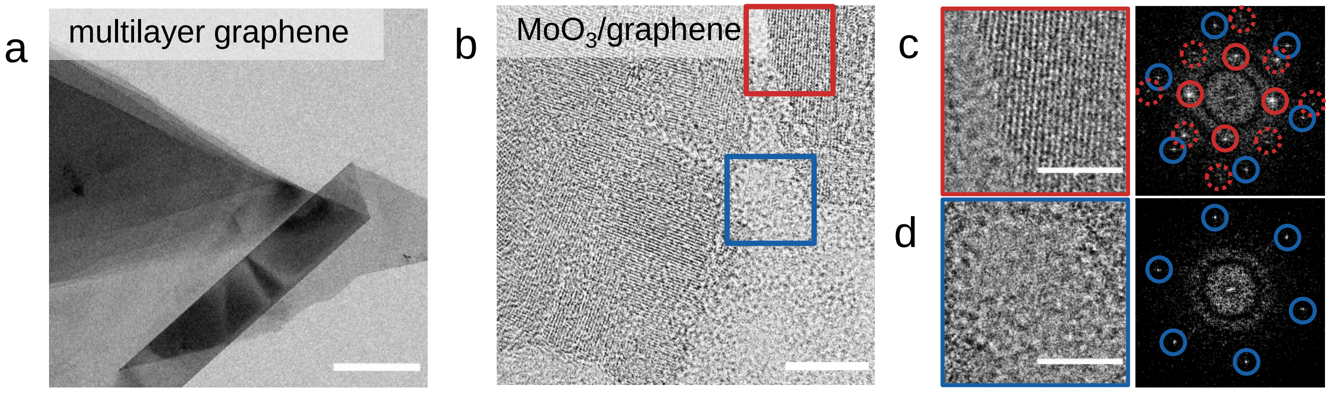

Figure S1: Atomic structure and topographical imaging of MoO_3 monolayer (1L) on multilayer graphene.
(**a**) low-magnification plan view TEM image of graphene nanosheet with MoO_3 flakes shown in HR-TEM image on (**b**). (**c** and **d**) zoom-in images of square regions as indicated by respective colors in (b) together with calculated FFT. The patterns of MoO_3 and HOPG are circled, respectively, in red and blue; while solid and dashed circles indicate first and higher order diffraction spots. The scale bars of (a), (b), and (c, d) are 200, 10, and 5 nm, respectively. (c) is introduced in Figure 1d-e in the main text.

Kelvin probe force microscopy (KPFM) characterisation

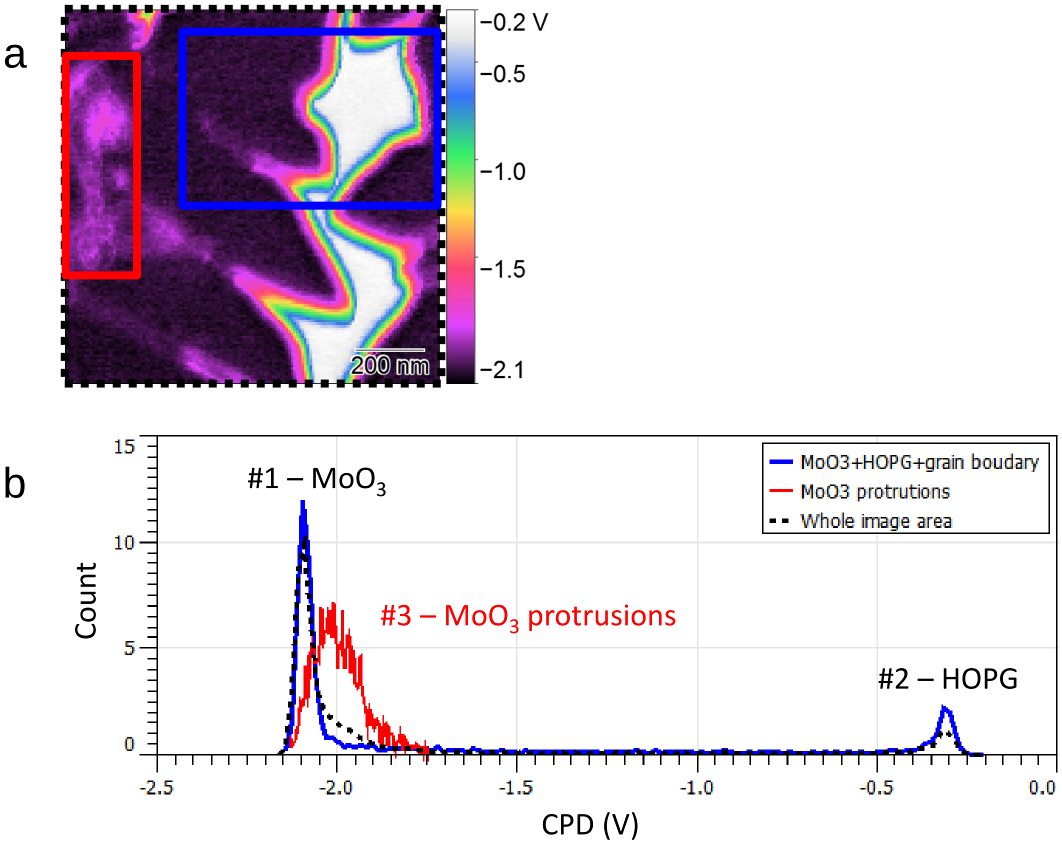

Figure S2: Local work function of MoO_3 monolayer (1L) on HOPG: KPFM surface potential measurement in UHV. (**a**) CPD map of 1L MoO_3 film on HOPG, same as in Figure 2b. White/blue color of CPD signal corresponds to a low WF (HOPG region), whereas pink/black corresponds to higher WF (MoO_3 region). (**b**) histograms extracted from three marked areas in (a):

I. MoO_3 and HOPG, including MoO_3 grain boundaries (blue line)
II. MoO_3 topographic defects (red line)
III. entire map (black, dash line)

As described in the main text, the above histograms show that MoO_3 and HOPG regions contribute to two distinguished surface potentials with relatively low distribution of 0.03 eV – see peaks #1 and #2 ascribed to MoO_3 and HOPG, respectively. The histograms extracted from two considered areas I and III show those peaks. Area III, covering the entire CPD map has additional counts at -2.0 V, which is clear when comparing histograms from I and III. Structural defects (area II) have slightly lower WF, which on histogram gives rise to an additional broader component, which is located at higher CPD of 0.1 V than smooth MoO_3, i.e., lower WF.
Therefore, the considered three histograms highlight three peaks:

#1 assigned to MoO_3
#2 assigned to HOPG
#3 assigned to MoO_3 topographic defects.

Kelvin probe force microscopy (KPFM) characterisation

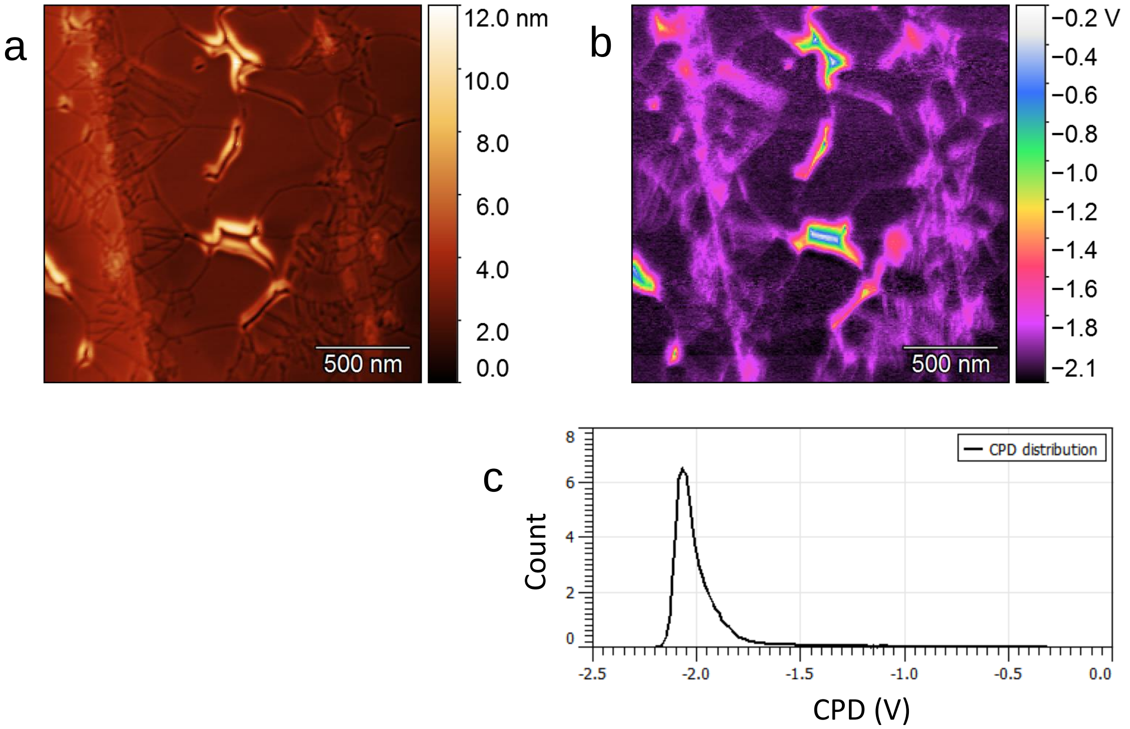

Figure S3: Local work function of MoO_3 monolayer (1L) on HOPG: KPFM surface potential measurement in UHV. (**a**) topography of 1L MoO_3 film with multiple grains of various sizes on HOPG, where MoO_3 edges are artificially lifted. (**b**) corresponding CPD map. White/blue color of CPD signal corresponds to a low WF (HOPG region), whereas pink/black corresponds to higher WF (MoO_3 region). (**c**) histogram extracted from (b) showing a peak at -2.1 V.

The KPFM measurements show that the high work function (WF) of MoO_3 monolayer is achieved independently of the grain size. For small grains and big grains, a plateau of CPD is observed at the same level. We observed slightly lower WF at the grain boundaries (highlighted by saturated pink), however, the difference is only < 0.1 V at these boundaries.

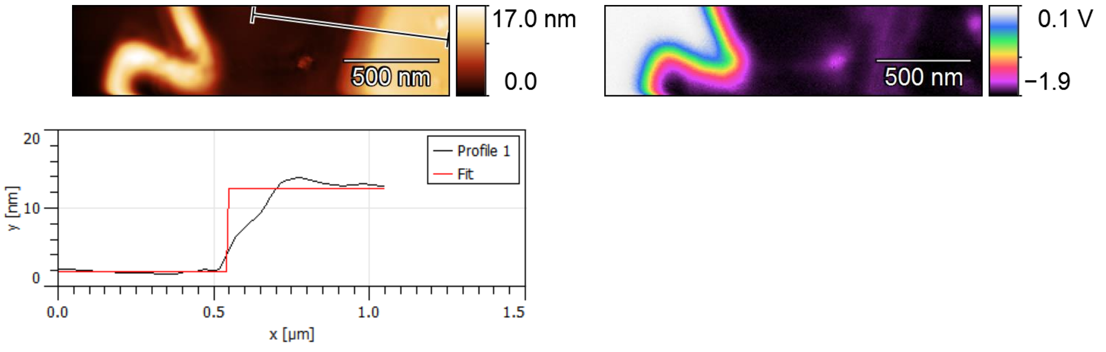

Figure S4: Local work function of MoO_3 monolayer on HOPG: KPFM in UHV. KPFM surface potential measurement of monolayered MoO_3 islands on HOPG substrate (partially uncovered). Left: topography; right: corresponding contact potential difference map. White/blue color of CPD signal corresponds to a low WF (HOPG region), whereas pink/black corresponds to higher WF (MoO_3 region). Down: a line scan through the topography shows HOPG steps of height ~11 nm covered by MoO_3 films.

There is no CPD differences on both HOPG terraces separated by 11-nm-step which proves that measured CPD is independent of topographical height variations in our measurement.

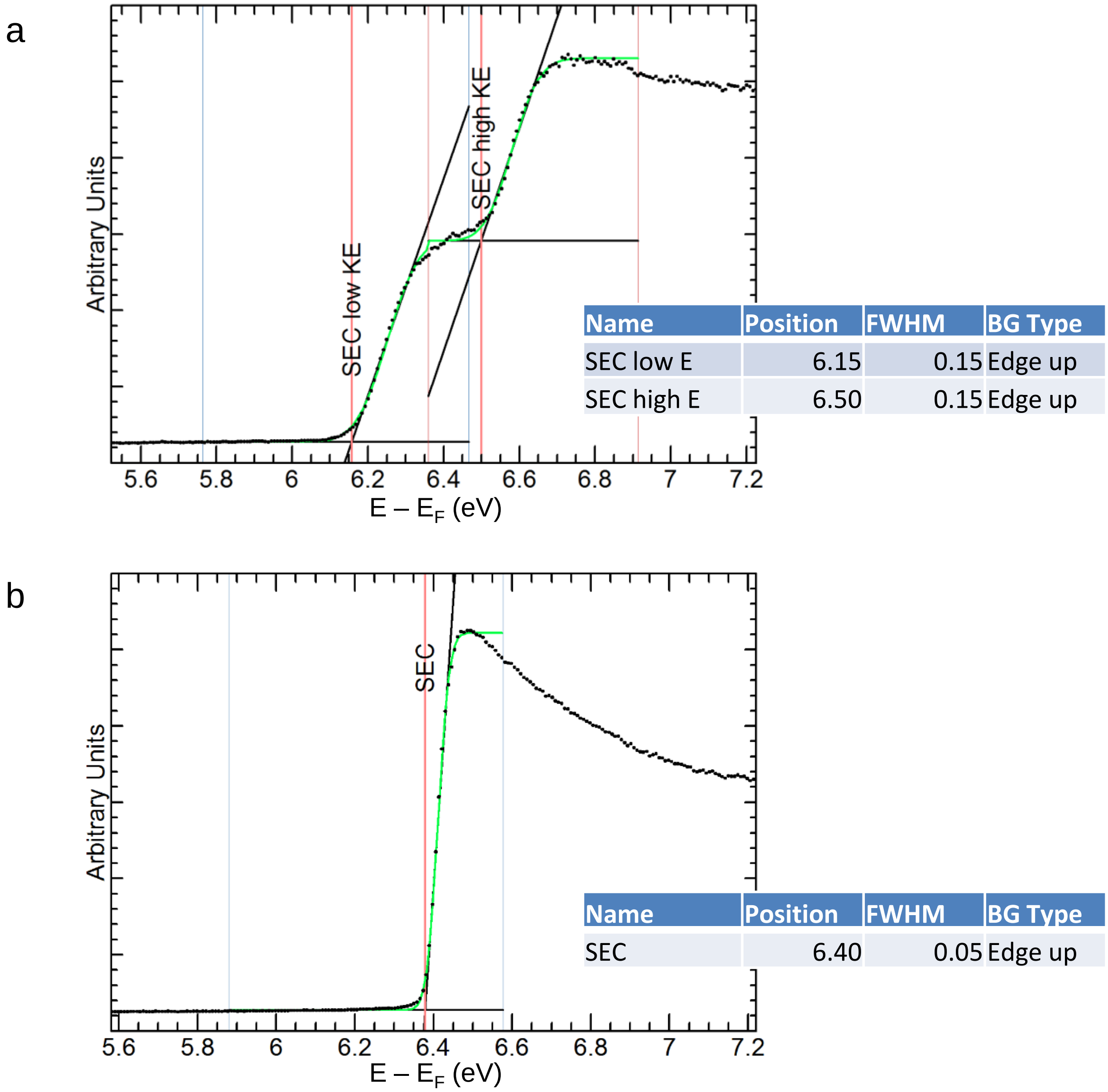

Name	Position	FWHM	BG Type
SEC low E	6.15	0.15	Edge up
SEC high E	6.50	0.15	Edge up

Name	Position	FWHM	BG Type
SEC	6.40	0.05	Edge up

Figure S5: Intersection measurements in secondary electron cutoff (SEC) region of the He I UPS spectra. For fitting presentation, replotted spectra of the samples introduced in the main text, in Figure 3b-c : (**a**) a multicomponent surface: uncovered HOPG patches and top monolayer MoO_3 islands, and (**b**) coalesced monolayer MoO_3 film. Modeling a step edge is performed in CasaXPS using the Edge up background type.

Ultraviolet photoelectron spectroscopy (UPS) characterisation

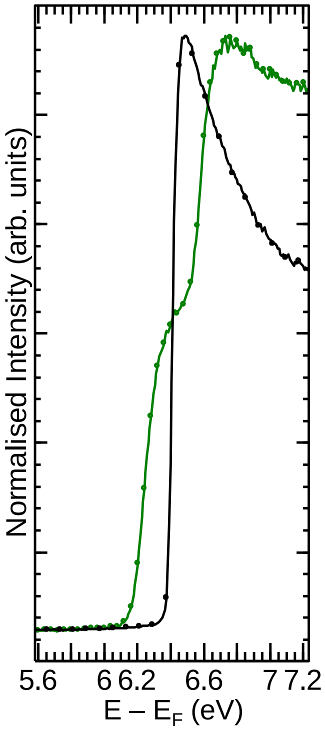

Figure S6: Secondary electron cutoff (SEC) region of the He I UPS spectra of a multicomponent surface: uncovered HOPG patches and top monolayer MoO_3 on HOPG. Replotted for direct comparison, overlaid spectra of the samples introduced in the main text, in Figure 3b-c. The data is displayed in normalized intensity to highlight different shapes of spectra, which depend on the area ratio between MoO_3 islands and the HOPG substrate.

Density-functional theory (DFT) calculations of electrostatic potential

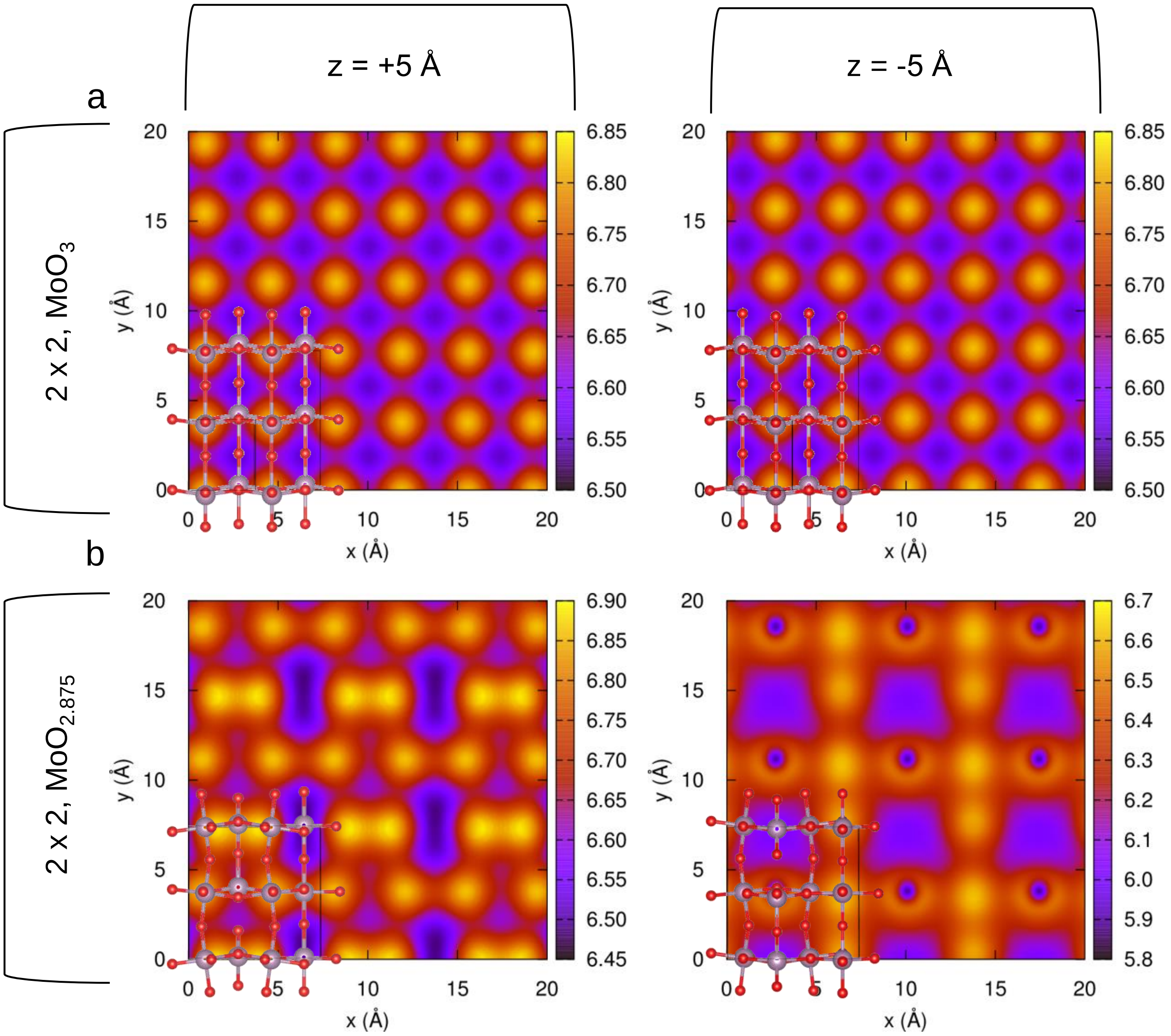

Figure S7: DFT calculations of electrostatic potential of monolayer: (**a**) stoichiometric MoO_3(010) and (**b**) defective $MoO_{2.875}$(010). Maps of work function (in eV) over the surface of MoO_3 and $MoO_{2.87}$ in two positions in the *z*-direction: z = -5 Å and z = 5 Å from the oxygen atoms at the edge of monolayer. If the z distance increases, then the variation of the work function with position above the plane decreases – until eventually everything smooths out and the value from the graph (Figure 5 in the main text) remains. The top views of atomic structures of considered monolayers are given as insets to correlate with work function distribution.

Note that for stoichiometric MoO_3 work function maps from two sides of the monolayer are as expected the same. While for $MoO_{2.875}$ the maps for the upper (without defect) and bottom (with O vacancy) sides are altered significantly.
